# Subnanosecond Charge Recombination Dynamics in P3HT/PC_61_BM Films

**DOI:** 10.3390/molecules171213923

**Published:** 2012-11-23

**Authors:** Wei Zhang, Ning-Jiu Zhao, Ming-Ming Huo, Li-Min Fu, Xi-Cheng Ai, Jian-Ping Zhang

**Affiliations:** 1 Center for Condensed Matter Science and Technology, Department of Physics, Harbin Institute of Technology, Harbin 150001, China; Email: zhang_wei_hit@126.com; 2 Department of Chemistry, Renmin University of China, Beijing 100872, China; Email: njzhao2010@163.com (N.-J.Z.); hithuomm@163.com (M.-M.H.); lmfu@chem.ruc.edu.cn (L.-M.F.); xcai@chem.ruc.edu.cn (X.-C.A.)

**Keywords:** [6,6]-phenyl-C_61_-butyric acid methyl ester (PCBM), poly(3-hexylthiophene) (P3HT), solvent vapor annealing (SVA), time-resolved spectroscopy, bimolecular charge recombination, trap states

## Abstract

Ultrafast near-infrared absorption spectroscopy was used to investigate the influence of film morphology and excitation photon energy on the charge recombination (CR) dynamics in the initial nanosecond timescale in the P3HT/PC_61_BM blend films. With reference to the CS_2_-cast films, the solvent vapor annealed (SVA) ones show 2–3-fold improvement in hole mobility and more than 5-fold reduction in the polymer-localized trap states of holes. At *Δt* = 70 ps, the hole mobility (*μ*_h_) and the bimolecular CR rate (*γ*_bi_) of the SVA films are *μ*_h_ = 8.7 × 10^−4^ cm^2^·s^−1^·V^−1^ and *γ*_bi_ = 4.5 × 10^−10^ cm^3^·s^−1^, whereas at *Δt* = 1 ns they drop to 8.7 × 10^−5^ cm^2^·s^−1^·V^−1^ and 4.6 × 10^−11^ cm^3^·s^−1^, respectively. In addition, upon increasing the hole concentration, the hole mobility increases substantially faster under the above-gap photoexcitation than it does under the band-gap photoexcitation, irrespective of the film morphologies. The results point to the importance of utilizing the photogenerated free charges in the early timescales.

## 1. Introduction

The continuing research efforts on bulk heterojunction (BHJ) polymer solar cells have recently achieved a power conversion efficiency (PCE) over 8%, benefiting from the advances in narrowband semiconducting copolymers and the morphological optimization of the photoactive layers [1,2,3]. Poly(3-hexylthiophene) (P3HT) as both light harvestor and electron donor (D) combined with [6,6]-phenyl-C_61_-butyric acid methyl ester (PC_61_BM) as electron acceptor (A) maintain a PCE of ~5% [4,5,6], which to date is still the highest among the broadband polymer devices. The P3HT/PC_61_BM photoactive layer is unique in its phase separation, *i.e.*, the polymer phase self-assembles into nano-fibrillar crystallites with stacked lamellar structures [7,8,9]. Differing from P3HT, the narrowband polymer in the photoactive layer is organized in a relatively disordered manner. In either cases it is the large area of D-A interfaces that facilitate the dissociation of neutral or charge transfer (CT) excitons, and thereby substantially boost the efficiency of charge photogeneration [10]. However, the large interfacial area also raises the probability of unwanted charge recombination (CR), which in competition with charge separation determine the yield of free charges. In addition, CR is a major photocurrent loss mechanism involving in the processes of charge transport. Accordingly, morphological optimization of the BHJ layer with various protocols of post-deposition annealing [4,5,11,12,13], aiming at minimizing the traps and recombination centers of free charges besides improving the carrier mobilities, has been shown to be crucial in reducing the CR probability [14,15,16]. The morphological optimization also provides an overall compromise between exciton dissociation and charge transport, which ensures the efficient utilization of the primary photogenerated free carriers.

Two different types of CR reactions, namely, the geminate and the bimolecular ones, have been attracting much attention [15]. The former taking place between a pair of hole and electron born from the same electronic excitation, *i.e.*, the bound polaron pair at the D-A interface [14], is independent on charge concentration, whereas the latter occurring between a pair of free hole and electron is concentration dependent. Analyses of the current-voltage characters of polymer solar cells show that bimolecular CR is an important loss mechanism of free charges [17,18]. However, other studies suggest that the bimolecular CR rate is several orders of magnitude lower than that predicted by the Langevin model and, consequently, the CR loss may be less important [19,20,21]. Therefore, a consensus with the contribution of bimolecular CR to the photocurrent loss has not been reached.

The bimolecular CR dynamics of photogenerated charge carriers in μs-ms timeframes have been intensely investigated by the use of transient absorption (TA) spectroscopy [16,19,22,23,24,25,26]. According to the trap-filling model, the faster decay component in the μs timescale of the hole kinetics originates from the recombination of *free* holes that are energetically below the edge of de-trapping (the bottom of the exponential distribution of trap states), whereas the slower component arises from the recombination of *de-trapped* holes [16,19,23]. In addition, the temporal evolution of hole concentration can be described by the power law, which is suggested to be generic for the polymer/PCBM blend systems [16,23]. For the CR dynamics of P3HT/PC_61_BM film in the ns-μs regimes, recent studies succeeded in differentiating the bimolecular from the geminate CR processes, and the CT-exciton was found to live in a timescale of 1.5 ns with characteristic optical absorption in 750–850 nm [14,15]. In addition, the CR dynamics in the ps-ns regimes had also been intensely studied, which were analyzed by the use of the general exponential expression [14,27,28], the power law [29,30] and the correlated rate equations [31,32,33].

It is known that the excitation photon energy influences the diffusion of singlet exciton in the polymer phase [34,35], as well as the dissociation of bound polaron pairs at the D-A interfaces [36]. In addition, the polymer photoexcitation bears morphological selectivity, e.g., under 600 nm (2.1 eV) P3HT nanocrystallites are preferentially excited, whereas under 400 nm (3.1 eV) both disordered and crystalline P3HT phases are agitated [14]. Since the primary yield of charges relies on the competition of CR with ultrafast charge photogeneration, it is important to examine the effect of excitation photon energy on the CR dynamics especially in the subnanosecond regime.

The present work has attempted to investigate the effects of film morphology and excitation photon energy on the subnanosecond CR dynamics of the P3HT/PC_61_BM blends. To this end, we prepared the solvent vapor annealed (SVA) and the carbon disulphide (CS_2_) casted P3HT/PC_61_BM films, and examined the ultrafast hole dynamics by means of femtosecond near-infrared TA spectroscopy. The CR dynamics in ps-ns timeframes with varying the excitation fluence over nearly two orders of magnitudes were analyzed by the use of the power law within the framework of trap-limited hole transport. The bimolecular CR rate and the hole mobility were derived and compared to the literature values, and were found to be morphological and photon-energy dependent. In addition, the temporal evolution of the CR rate and the hole mobility provide a generalized picture for the somewhat scattered literature values, as well as a deeper insight into the photocurrent loss mechanisms.

## 2. Results and Discussion

### 2.1. Characterization of the Hole Dynamics in Subnanosecond Timeframe

**Figure 1:**
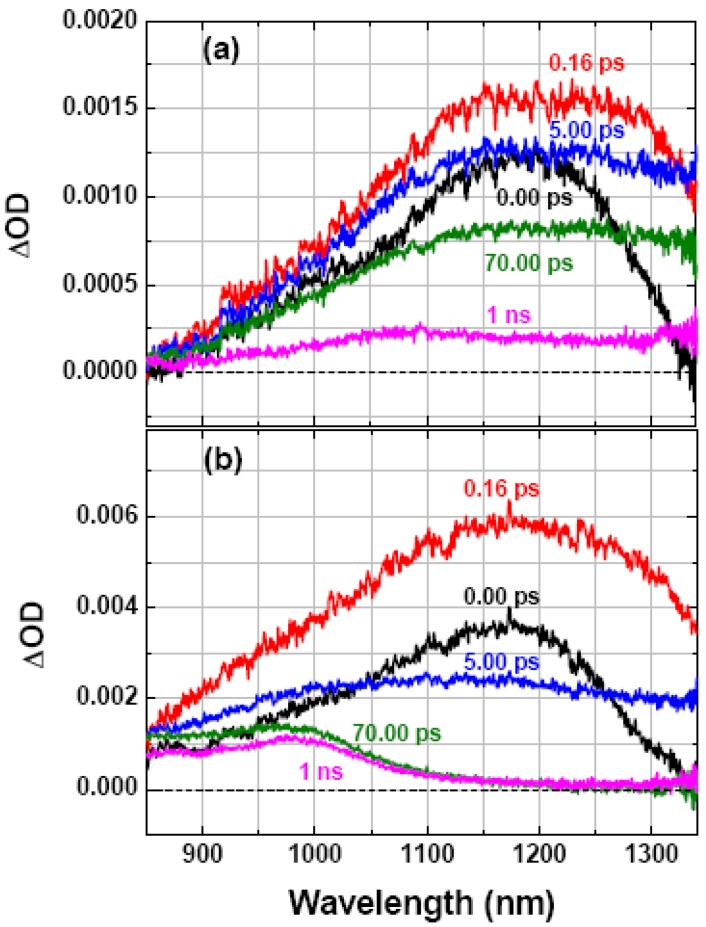
Transient spectra at selected delay times recorded following photoexcitation at 620 nm for (**a**) SVA neat P3HT film and (**b**) SVA P3HT/PC_61_BM blend film (1:1, w/w). Excitation photon fluence were 4.3 × 10^17^ and 7.8 × 10^17^ photons·cm^−3^·pulse^−1^ for (**a**) and (**b**) respectively.

Figure 1 shows that, immediately following the pulsed optical excitation at 620 nm (*Δt* = 0.0 ps) [37], the neat and the blend films exhibit broadband absorption peaking at ~1,180 nm, which are attributed to the excited state absorption of the lowest-lying singlet exciton (P3HT*). Notably, at *Δt* = 70 ps the exciton absorption, still sizable for the neat film (Figure 1a), decayed out completely for the blend film (Figure 1b). The accelerated exciton relaxation in the blend film is due to the dissociation of P3HT* at the D-A interface, which leads to the formation of cationic polaron P3HT·^+^ (hereafter referred to as *hole*) characterized by the reminiscent absorption in 850–1,150 nm at the delay time later than *Δt* = 70 ps [14,15]. In neat film, the band-gap excitation (620 nm, 2.0 eV) did not produce any appreciable amount of holes, in accordance with the photon-energy dependence of hole photogeneration, *i.e.*, the excess energy is needed for exciton dissociation [38]. To the contrary, the band-gap excitation of the blend film readily yielded holes via either instantaneous or diffusion-limited exciton dissociation [14,39]. Note that the singlet exciton annihilation may come into play under sufficiently high photon fluence, leading to an extra volume of holes. However, the intrinsic hole dynamics becomes predominant after *Δt* = 70 ps, because the timescales of exciton annihilation via direct exciton contact or diffusion-limited exciton collision and that of diffusion-limited exciton dissociation are less than 10 ps.

**Figure 2:**
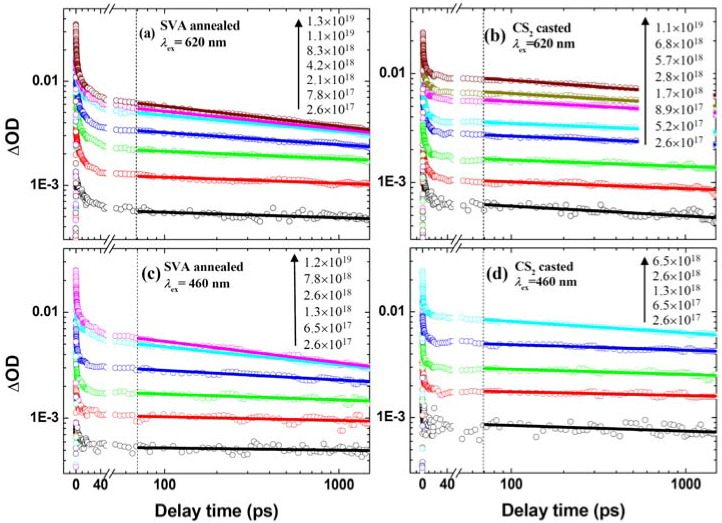
Semi-log plots of the kinetics probed at 1,000 nm under the indicated excitation wavelengths (*λ*_ex_) and photon fluences (in photons·cm^−3^·pulse^−1^) for (**a**, **c**) SVA and (**b**, **d**) CS_2_-cast P3HT/PC_61_BM blend films (1:1, w/w). Solid lines are the best fits to the power law, ΔOD(*t*) = *A*·*t*^−*α*^. Vertical dashed lines indicate the occurrence of the delay time of *Δt* = 70 ps.

Figure 2 shows the hole kinetics of P3HT/PC_61_BM films subjected to SVA or CS_2_-cast treatments. In the SVA blend film, P3HT is phase separated from PC_61_BM via nanoscale crystallization, whereas in the CS_2_-cast blend film the phase separation is rather poor owing to the fast drying process of CS_2_. The distinct morphological difference has been confirmed by the use of atomic force microscopy (AFM) and X-ray diffractometry, showing a much higher crystallinity of the P3HT phase upon the SVA post-deposition treatment [39]. In addition, the device performance including the current-voltage characteristics, the filling factor, the external quantum efficiency (EQE) and the PCE are much higher upon the SVA post-deposition treatment (Supporting Information S1). Therefore, these two different types of preparations provide the morphological contrast for comparing the CR dynamics. Here, it is important to note that the optical probe at 1,000 nm preferentially detects the localized holes [14,38], providing an opportunity to examine the holes localized to the disordered polymer phases, where the traps and the recombination centers of holes are concentrated.

### 2.2. Effects of Film Morphology and Excitation Photon Energy/Fluence on the Hole Dynamics

For the SVA blends under either 620 nm or 460 nm photoexcitation, the increase of ΔOD amplitudes become saturated when the photon fluences exceed 8.3 × 10^18^ photons·cm^−3^·pulse^−1^ (Figure 2a,c). However, the saturation effects are much less evident in the cases of CS_2_-cast films (Figure 2b,d). Since the SVA treatment increases the mobility and the delocalization extent of P3HT* by improving the crystallinity of the P3HT phase, the exciton annihilation taking place in the initial tens of picoseconds is expected to be more efficient than that in the CS_2_-cast films. This is responsible for the different saturation behavior between the two types of blends. On the other hand, as seen from the hole kinetics in Figure 2a, the population relaxation in 70–1,450 ps depends on the incident photon fluence, *i.e.*, the decay gets faster on going from 10^17^ to 10^19^ photons·cm^−3^·pulse^−1^. The subnanosecond decay of the 1,000-nm kinetics must be due to the bimolecular CR reaction of P3HT·^+^ with PC_61_BM·^−^, because the geminate CR reaction of bound polaron pair, best probed at the characteristic absorption in 750–850 nm [14], is independent on photon fluence. At the lowest excitation fluences, the ΔOD amplitudes for the SVA blends drop for 5–10% from Δ*t* = 70 ps to 1 ns (Figure 2a,c), whereas those for the CS_2_-cast blends drop for 15–20% (Figure 2b,d), indicating the significant reduction of the hole recombination centers upon SVA treatment. In addition, a higher excitation photon energy (*λ*_ex_ = 460 nm) results in a slightly slower decay of the hole kinetics regardless of the film morphologies.

**Figure 3:**
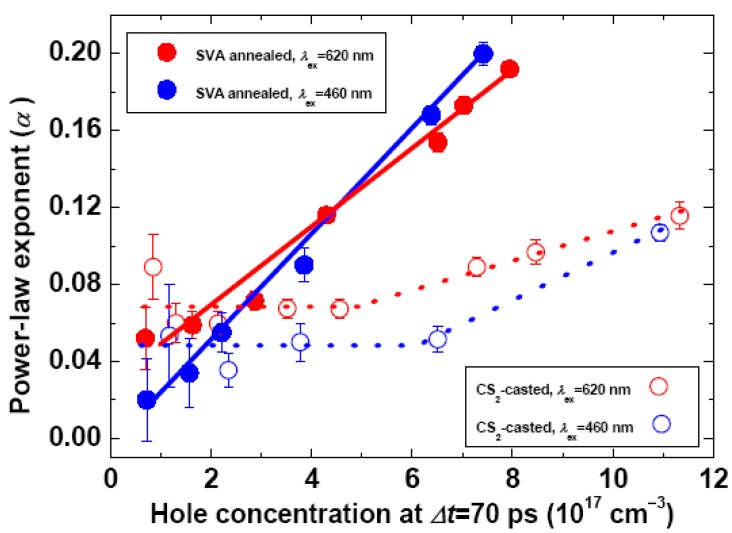
Plot of power-law exponent against the hole concentration (the *α*-plot) at *Δt* = 70 ps for SVA and CS_2_-cast P3HT/PC_61_BM blend films (1:1, w/w) under the excitation wavelengths of 620 nm or 460 nm. The hole concentration were calculated from the ΔOD amplitudes at *Δt* = 70 ps, for which the extinction coefficient of hole at 1,000 nm and that of exciton at 1,200 nm were assumed to be the same [38]. Solid and dashed lines are for guiding the eyes.

The hole kinetics spanning 70–1,450 ps in Figure 2 fit nicely to the power law, ΔOD(*t*) = *A*·*t*^−^*^α^*, which is known to originate from the bimolecular recombination in the presence of an exponential distribution of the polymer-localized trap states (PLTSs) in energetically disordered solids [16,40]. The applicability of the power law may be justified by the log-log plots of the hole kinetics in Figure S2 showing that later (earlier) than Δ*t* = 70 ps the recombination kinetics appear as (deviate from) straight lines, which are especially evident at relatively lower excitation fluences. The success in describing the decay behavior of holes by using the power law supports the trapping-filling model for hole recombination. In the μs-ms timeframes, the exponent *α* is a criterion of localized (*α*~0.5) or delocalized (*α*~1) hole within the framework of trap limited hole transport [24]. However, from Figure 3 depicting the plot of exponent against hole concentration (the *α*-plot), we seen that the *α* values are generally below 0.2, which are considerably smaller than those derived from the μs-ms kinetics in literatures (0.25–0.65) [19,41]. This together with the preference of the 1000-nm kinetics in probing the localized holes suggest that, in the subnanosecond regime of interests, the parameter *α* cannot be an absolute measure of the extent of hole delocalization, rather, it should be regarded as a potentiality index of hole mobility.

Recent TA studies demonstrated that thermal annealing of the P3HT/PC_61_BM blend promotes the crystallinity of the polymer phase, resulting in the increase of *α* for the hole kinetics in the μs-ms regimes [16]. We now examine the morphological effects on the subnanosecond CR dynamics characterized by the *α*-plots in Figure 3. It is seen that there exists a concentration range for the CS_2_-cast film, ≤5 × 10^17^ cm^−3^, where *α* remains constant, whereas the range of constant *α* for the SVA film is much lower, ≤1 × 10^17^ cm^−3^. According to the trap-filling model, holes of low concentration tend to be trapped by the PLTSs. After filling up the PLTSs, extra holes become mobile via occupying the valence band states of the semiconducting polymer. In addition, holes in shallow PLTSs can be librated via thermal activation. In these ways, holes become free, and *α* increases upon further increasing hole concentration. This is illustrated clearly by the inflexion points in Figure 3, which are especially evident for the CS_2_-cast films. The inflexion points can be taken as the thresholds of filling-up PLTSs, and hence the corresponding hole concentration represents the PLTS density, which turns out to be ~5 × 10^17^ cm^−3^ and ≤1 × 10^17^ cm^−3^ for the CS_2_-cast and the SVA blends, respectively. The PLTS densities thus derived are robust, because at *Δt* = 1 ns the *α*-plots give rise to similar results within experimental errors (Figure S3), *i.e.*, the PLTS density should be intrinsic to material and hence be independent on delay time. Note that the PLTS density of CS_2_-cast blend agrees well with that of the unannealed P3HT/PC_61_BM blend determined by using μs-ms TA spectroscopy (7 × 10^17^ cm^−3^) [16].

Previous studies showed that in μs-ms the CR dynamics of MDMO-PPV/PC_61_BM blend was unaffected by post-deposition annealing [23]. However, thermal annealing of the P3HT/PC_61_BM blend significantly shallowed and concomitantly increased the trap states [16,42]. Our results prove that the SVA treatment reduces the PLTS density to one fifth of that of the CS_2_-cast blend. For an open-circuit P3HT/PC_61_BM solar cell under the 1 sun terrestrial irradiation, the hole concentration in the BHJ layer is on the order of ~10^17^ cm^−3^ [24,43]. The significant minimization of PLTSs by SVA treatment implies that, under natural light irradiation, a large fraction of holes in the SVA film remain free after filling up the PLTSs, whereas the holes in the CS_2_-cast film are mostly trapped by the PLTSs. These results corroborate to the significantly enhanced PCE of the P3HT/PC_61_BM device fabricated via combined SVA and thermal annealing [4,12,44].

In the low hole concentration regime (Figure 3), *i.e.*, below the inflexion points of the *α*-plots, the *α* values are rather small (<0.08) under either 460 nm or 620 nm photoexcitation. This is also seen from the hole kinetics under low photon fluence in Figure 2, indicating that at *Δt* = 70 ps holes are partially mobile, *i.e.*, not fully trapped by the PLTSs. This phenomenon is interpreted in terms of the *prevention effect* of P3HT crystallite [16]: A small fraction of holes besieged in the P3HT crystallites remain mobile until being trapped by the PLTSs and recombined therein. It had been shown that the PLTSs mainly inhabit the disordered P3HT phase, and that photoexcitation at 620 nm and 460 nm, respectively, preferentially excite the crystalline and the disordered P3HT phases [14,24]. Consequently, the photoexcitation at 620 nm leads to less filled PLTSs and relatively more mobile holes capable of bimolecular CR, which readily explains the ~40% higher *α* values than those under the photoexcitation at 460 nm irrespective to film morphology (Figure 3). In spite of the relatively low red-edge absorptivity of the P3HT/PC_61_BM solar cell, the internal quantum efficiency (IQE, 70–76%) is nearly uniform across the light harvesting range of 460–625 nm [44]. This may be understood in view of the aforementioned photon-energy dependence of hole mobility under low hole concentration: Under the device operation condition, the band-gap compared to the above-gap excitation creates the crystallite-confined holes of higher mobility, which can be extracted with higher efficiency.

In the high hole concentration range (Figure 3), *i.e.*, above the inflexion points of the *α-*plots, photoexcitations at 460 nm or 620 nm for the SVA films lead to substantially larger *α* values and faster increase of *α* with reference to the CS_2_-cast films, implying that the SVA treatment effectively improves the hole mobility via minimizing the trap structures.

### 2.3. Concentration, Photon Energy and Time Dependence of Hole Mobility and Bimolecular Recombination

The kinetics under relatively higher photon fluences in Figure 2, corresponding to the hole concentration above the inflexion points in Figure 3, are to be used to derive the bimolecular CR rate constants, because mobile holes are capable of recombining with electrons, while those trapped in PLTSs can do so provided that they get free via thermal activation (activation energy, 50 meV [45]) [16,25]. The latter prerequisite of bimolecular CR is corroborated by the rather slow (modest) decay of hole kinetics under the lowest (higher) photon fluences as shown in Figure 2. The bimolecular CR rate (*γ*_bi_) can be written as (Supporting Information S4):

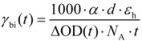
(1)
where, *N_A_* is Avogadro’s constant, *α* is the power-law exponent, ΔOD(*t*) represents the hole population (*cf* Figure 2), and *d* and *ε*_h_, respectively, denote the film thickness (*d* = 190 nm) and the extinction coefficient of hole (*ε*_h_=3 × 10^4^ M^−1^·cm^−1^) [24]. Following Langevin’s hole-limited model of bimolecular CR [17,18], the mobility of free hole (*μ*_h_) reads:

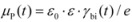
(2)
where *ε*_0_ and *ε*, respectively, stand for the vacuum permittivity and the dielectric constant of the P3HT/PC_61_BM blend (*ε* ~ 3.5) [42], and *e* is the elementary charge.

**Figure 4:**
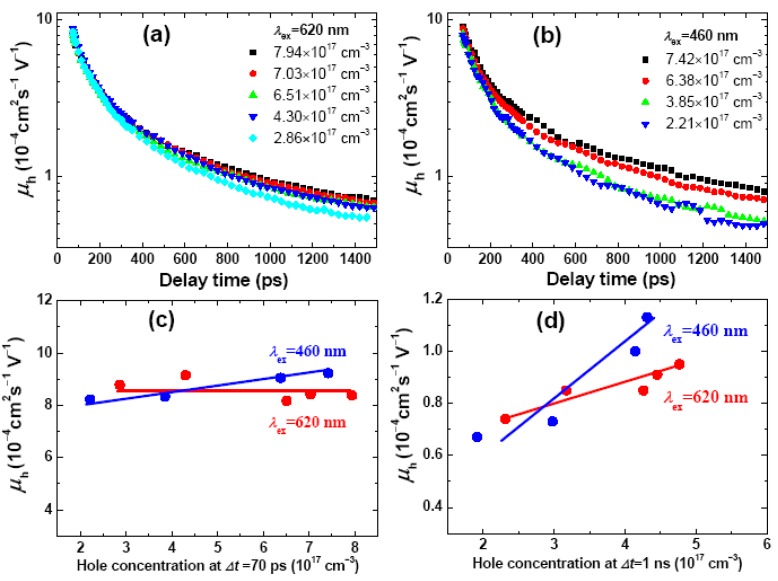
Temporal evolution profiles of hole mobility (*μ*_h_) for the SVA blend P3HT/PC_61_BM films (1:1, w/w) under photoexcitation at (**a**) 620 nm and (**b**) 460 nm. Hole concentration at *Δt* = 70 ps are indicated in each panel. Change of *μ*_P_ at (**c**) *Δt* = 70 ps and (**d**) *Δt* = 1 ns as a function of hole concentration. The excitation wavelengths (*λ*_ex_) are indicated in each panel. Solid lines are for guiding the eyes.

For the SVA blend films, Figure 4a,b show the temporal evolution of hole mobility under different excitation wavelengths as derived by the use of Equations (1) and (2). It is seen that, under the photoexcitation at 620 nm or 460 nm, the evolution profiles under various hole concentration become diverged as the delay time elapses. Such tendency is more clearly illustrated in Figure 4c,d: The hole mobility increases much faster at *Δt* = 1 ns than that at *Δt* = 70 ps, and in both cases it increases substantially faster under the 460 nm excitation than that under 620 nm (the bimolecular CR rates, *γ*_bi_, show similar temporal evolution. Figure S4, Supporting Information S5). From these results we draw the following conclusions: (i) *The PLTS filling up time*. The PLTSs are filled up approximately in a 100 ps timescale as evidenced by the delay time when the hole mobility starts to diverge. Specifically, the filling-up time under the 460 nm excitation (a few tens of picoseconds) seems considerably shorter than that under the 620 nm excitation (~200 ps); (ii) *The concentration dependence of hole mobility*. In Figure 4c before or about filling up the PLT Ss (*Δt* ~ 70 ps), the hole mobility, 8.6 × 10^−4^ cm^2^·s^−1^·V^−1^ (620 nm) and 8.7 × 10^−4^ cm^2^·s^−1^·V^−1^ (460 nm), do not vary appreciably upon changing hole concentration, in accordance with the previously reported concentration independence of hole mobility [16,24]. However, in Figure 4d, after filling up the PLTSs (*Δt* ~ 1 ns), the hole mobility becomes 8.6 × 10^−5^ cm^2^·s^−1^·V^−1^ (620 nm) or 8.8 × 10^−5^ cm^2^·s^−1^·V^−1^ (460 nm), although upon increasing the hole concentration it increases for 28% (620 nm) and 68% (460 nm); (iii) *The photon-energy dependence of hole mobility*. As manifested by the steeper rise of *μ*_h_ under the 460 nm excitation (Figure 4c,d), the above-gap excitation creates more mobile holes than the band-gap excitation does; (iv) Similar photon energy and hole concentration dependence of *μ*_h_ and *γ*_bi_ were observed for the CS_2_-cast blends (Figure S5, Supporting Information S5). Importantly, as seen from Table 1 the hole mobilities of the CS_2_-cast blends are 2–3 times lower than those of the SVA blends, manifesting the essential role of SVA in reducing the PLTSs.

Since the hole concentration of an open-circuit P3HT/PC_61_BM device under the 1 sun condition (~10^17^ cm^−3^) [24,43] is comparable to those at *Δt* = 70 ps or *Δt* = 1 ns in the low-photon-fluence laser experiments, our results on the hole dynamics are of practical implications. E.g., for a solar cell with a thickness of BHJ layer *d* = 100 nm and an open-circuit voltage *V*_oc_ = 0.6 V, and assuming a higher (lower) hole mobility as that at *Δt* = 70 ps (*Δt* = 1 ns), the timescale of hole collection is estimated to be *d*^2^/*μ*_h_·*V*_oc _≈ 190 ns (1.9 μs). In view of the quadratic dependence of hole collection time on *d*, a thinner BHJ layer is obviously facial for carrier extraction, however, this has to compromise with the efficiency of light harvesting, *i.e.*, requires polymers with even higher absorptivity. Such a scenario suggests the importance of efficiently utilizing the holes yielded in the initial temporal regime, as otherwise the efficiency of hole collection can be declined owing to the prolonged collection time. In a working device exposed to continuous white-light illumination, the hole mobility and hence its collection are dependent on the overall hole concentration induced by the light of a range of photon energy and, consequently, the deep enough PLTSs that cannot be thermally activated are mostly occupied. In addition, the effects of electrode and external electrical field on the CR dynamics have to be considered.

**Table 1:** Averaged hole mobility (*μ*_h_) and bimolecular CR rate (*γ*_bi_) under different excitation wavelengths (*λ*_ex_) at *Δt* = 70 ps and *Δt* = 1 ns (in parentheses). The average was taken over a number of independent determinations under different hole concentrations (*cf* Figure 4,S4).

*λ*_ex _		SVA annealed P3HT/PC_61_BM film			CS_2_-casted P3HT/PC_61_BM film	
(nm)		*μ*_h_ (cm^2^·s^−1^·V^−1^)	*γ*_bi_ (cm^3^·s^−1^)		*μ*_h_ (cm^2^·s^−1^·V^−1^)	*γ*_bi_ (cm^3^·s^−1^)
620		8.6 × 10^−4^	4.5 × 10^−10^		3.8 × 10^−4^	2.0 × 10^−10^
		(8.6 × 10^−5^)	(4.5 × 10^−11^)		(3.4 × 10^−5^)	(1.7 × 10^−11^)
460		8.7 × 10^−4^	4.5 × 10^−10^		3.1 × 10^−4^	1.6 × 10^−10^
		(8.8 × 10^−5^)	(4.6 × 10^−11^)		(2.8 × 10^−5^)	(1.4 × 10^−11^)

We now compare the hole mobilities of the SVA blend films to the documented values. We have shown that the hole mobility is both concentration and photon energy dependent and, at the same time, is time-dependent. In addition, some of other experimental means for hole mobility determination are under non-zero electrical field. It is therefore not surprising to see that different methods render scattered results (see *Supporting Information S6* for a summary). Among the time resolved measurements, the hole mobility of 8.7 × 10^−4^ cm^2^·s^−1^·V^−1^ (*Δt* = 70 ps) and 8.7 × 10^−5^ cm^2^·s^−1^·V^−1^ (*Δt* = 1 ns) obtained in the present work agree well with those determined via time-of-flight (TOF) measurement in the 100 ns–1 ms timeframe (5 × 10^−5^–1 × 10^−4^ cm^2^·s^−1^·V^−1^) [12,46,47], and also agree with the result of space-charge-limited-current (SCLC) measurement (2 × 10^−4^ cm^2^·s^−1^·V^−1^) [48]. In addition, those assessed by the use of μs-ms TA spectroscopy are on the order of 10^−7^–10^−6^ cm^2^·s^−1^·V^−1 ^[19], which seems as an extrapolation of the temporal evolution of *μ*_h_ in Figure 4. It had been shown that the bimolecular CR rates in the μs~ms timeframes are about several orders of magnitude lower than that calculated based on Langevin analysis [19,20,21]. The present work shows that on going from *Δt* = 70 ps to *Δt* = 1 ns, the bimolecular CR rate drops for 1 order of magnitude, and on further going to the *μ*s~ms regimes, additional reduction in the bimolecular CR rate is expected. Taken together, our results prove that, in the subnanosecond regime and under relatively high excitation photon fluence, the hole dynamics can be well accounted for by Langevin’s model of hole-limited bimolecular CR.

## 3. Experimental

Regioregular poly(3-hexylthiophene) (RR-P3HT, head-to-tail > 90%, *M*_w_=43462, *M*_w_/*M*_n_=2.9) and PC_61_BM (~99%) were used as received from Aldrich (Saint Louis, MO, USA). For film preparation, neat P3HT or mixed P3HT/PC_61_BM (1:1, wt) were dissolved in *o*-dichlorobenzene (*o*-DCB) or CS_2_ to obtain the solutions of 20 mg·mL^−1^ (2%, wt). Quartz substrates were treated by ultrasonication in detergent, and washed successively with deionized water, acetone, ethanol and isopropyl alcohol, and on which the neat P3HT or the blend P3HT/PC_61_BM films were spin-coated (1,000 rpm; 30 s). The SVA treatment was performed by keeping the substrate coated from the *o*-DCB solution in Petri dishes under the *o*-DCB atmosphere for 1 hour. The films coated from the CS_2_ solutions were dried in open atmosphere to obtain the un-annealed photoactive layer referred to as the CS_2_-casted film. The preparations were conducted in a glovebox filled with argon (oxygen concentration below 0.1 ppm). The typical thickness of the photoactive layer was 190 nm as determined with an Alpha-Skep surface profiler (KLA-Tencor, Milpitas, CA, USA).

The TA apparatus with a temporal resolution of 160 fs is briefly described below. An optical parametric amplifier (OPA-800 CF-1, Spectra Physics, Mountain View, CA, USA) pumped by a regenerative amplifier (SPTF-100F-1KHPR, Spectra Physics) provided the actinic laser pulses at desired wavelengths (~120 fs, full width at half maximum). The continuum probe (800–1,400 nm) generated from a 3-mm thick sapphire plate was detected after interrogating the excited sample by an InGaAs detector (OMA-V, Princeton Instruments, Trenton, NJ, USA) attached to individual imaging spectrographs (SpectraPro 2300i, Princeton Instruments, Trenton, NJ, USA). To ensure that each laser shot excites the sample fully relaxed form the previous excitation, the laser system was run at a repetition rate of 333 Hz. To prevent the films from photodegradation, the films sandwiched with quartz slices were kept in vacuum. A mechanical chopper (Model 75158, Newport, Stratford, CT, USA) was set in the pump beam to regulate pump “on” and “off” for a pair of sequential actinic pulses. A magic-angle scheme was used on the pump-probe measurement. To improve the signal-to-noise ratio, each transient spectrum was obtained by averaging 200 individual measurements, and the typical detection sensitivity of the difference absorption (ΔOD) was better than 10^−4^. The time-resolved absorption spectra were corrected against group velocity dispersion. All measurements were carried out at room temperature (296 K).

## 4. Conclusions

We have demonstrated that, with reference to the CS_2_-cast, the SVA treatment improves the hole mobility 2–3 fold and reduces the PLTS density more than 5 fold for the P3HT/PC_61_BM blend. At *Δt* = 70 ps, the hole mobility and bimolecular CR rate for the SVA blend are *μ*_h_=8.7 × 10^−4^ cm^2^·s^−1^·V^−1^ and *γ*_bi_=4.5 × 10^−10^ cm^3^·s^−1^, in agreement with the literature values determined by TOF measurements, but 2–3 orders of magnitude larger than those obtained with the μs-ms TA spectroscopy. However, at *Δt* = 1 ns, they drop to *μ*_h_=8.7 × 10^−5^ cm^2^·s^−1^·V^−1^ and *γ*_bi_=4.6 × 10^−11^ cm^3^·s^−1^, approaching the μs-ms TA results. The analyses of the subnanosecond hole dynamics provide a generalized picture for the considerably scattered literature values of the bimolecular CR rate. Furthermore, upon increasing the hole concentration the hole mobility increases substantially faster under the above-gap photoexcitation than it does under the band-gap photoexcitation irrespective to the film morphologies. From *Δt* = 70 ps to 1 ns the hole mobility decreases ~90% despite a small reduction in the hole concentration (5–10%). Therefore, it is important to utilize the photogenerated charge carriers in early timescales, which may be realized by the optimization of film morphology and device configuration.

## Supplementary Materials

Electronic Supplementary Information (ESI) can be accessed at: http://www.mdpi.com/1420-3049/17/12/13923/s1. Data on device performance, power-law exponent plot, quantitative analyses of hole mobility and bimolecular CR rate, and summary of documented hole mobilities.
