# Supplementary material for: Subnanosecond Charge Recombination Dynamics in P3HT/PC61BM Films
Source: Molecules. 2012 Nov 23;17(12):13923–36. doi: 10.3390/molecules171213923 (PMC6268940; doi:10.3390/molecules171213923)
Supplement: Supplementary file 1 [file molecules-17-13923-s001.pdf]

Supporting Information

S1. Comparison of the Performance between the SVA and the CS_2-cast Treated Devices

The polymer solar cells were manufactured on patterned ITO-coated glass substrates. The ITO surface was modified by spin-coating PEDOT:PSS (Baytron P VP A1 4083) with a thickness of ~40 nm, followed by baking in air at 150 °C for 15 min. Photoactive layers were obtained by spin-coating the blend solution (1,000 rpm, 30 s). The SVA treatment was performed by keeping the substrate coated from the *o*-dichlorobenzene (*o*-DCB) solution of P3HT-$PC_{61}BM$ mixture in Petri dishes under the *o*-DCB atmosphere for 1 h. The films coated from the CS_2 solutions were dried in open atmosphere to obtain the un-annealed photoactive layer (referred to as the CS_2-casted film). The cathode consisted of 20 nm of calcium and 100 nm of aluminum, which were thermally evaporated to the top of the photoactive layer with a shadow mask to define an active area of 0.04 cm^2. The current-voltage curves were measured under 100 mW/cm^2 standard AM 1.5 G spectrum using a solar simulator (XES-70S1, San-Ei Electric Co. Ltd., Osaka, Japan); AAA grade, 70 × 70 mm^2 photo-beam size), for which a 2 × 2 cm^2 monocrystalline silicon cell (SRC-1000-TC-Q; VLSI Standards Inc., Milpitas, CA, USA) was used for reference. All the electrical measurements were performed in a nitrogen-filled glove box at room temperature.

Figure S1. (**a**) *I-V* characteristics of SVA and CS_2-casted P3HT/$PC_{61}BM$ devices under the illumination of AM 1.5G from a solar simulator (100 mW/cm^{-2}). (**b**) EQE curves of the corresponding polymer solar cells.

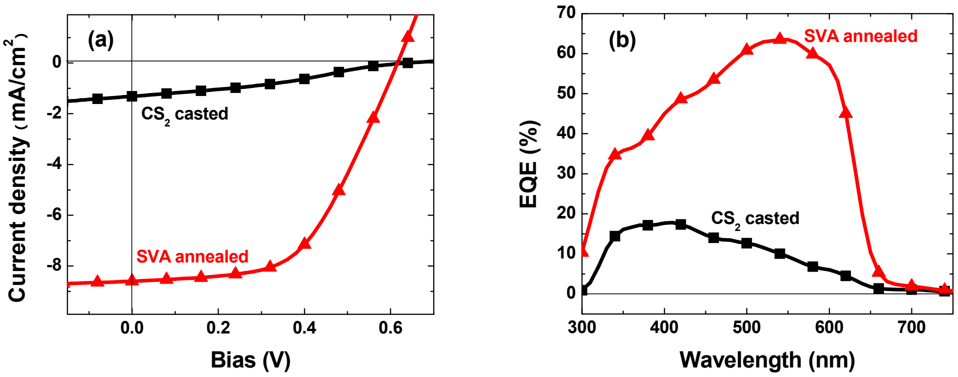

Table S1. Photovoltaic properties of SVA and CS_2-casted P3HT/$PC_{61}BM$ devices.

Devices	V_{oc}(V)	J_{sc} (mA/cm^2)	*FF* (%)	PCE (%)
P3HT/PCBM (CS_2 casted)	0.64	1.31	31.9	0.27
P3HT/PCBM (SVA annealed)	0.62	8.60	54.1	2.86

S2. Log-log Plots of Recombination Kinetics

Figure S2. Log-log plots of the kinetics probed at 1,000 nm under the indicated excitation wavelengths (λ_{ex}) and photon fluences (in photons·cm^{-3}·pulse^{-1}) for the SVA blend film of P3HT/PC$_{61}$BM (1:1, w/w). Solid lines are the best fits to the power law, $\Delta OD(t) = A \cdot t^{-\alpha}$. Vertical dashed lines indicate the occurrence of the delay time of Δt = 70 ps. (*cf* Figure 2a of the main text.)

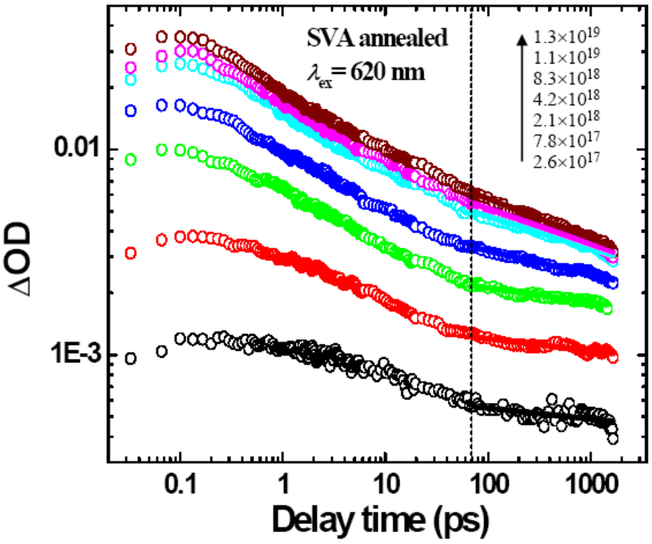

S3. Power-law Exponent Plot Against Hole Concentration (the α-Plot)

Figure S3. Plot of power-law exponent ($\Delta OD(t) = A \cdot t^{-\alpha}$) against the hole concentration at Δt = 1 ns for the SVA and the CS_2-casted blend P3HT/PC$_{61}$BM films (1:1, w/w) under the excitation wavelengths of 620 nm or 460 nm. The hole concentration were calculated from the ΔOD amplitudes at Δt = 1 ns, for which the extinction coefficients of hole at 1,000 nm and of exciton at 1,200 nm were assumed to be the same [S1]. The solid and the dashed lines are for guiding the eyes.

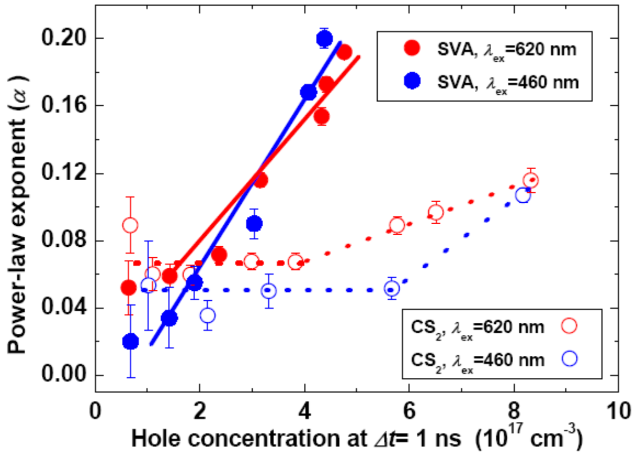

S4. Bimolecular Recombination Rate and Mobility of Hole

The rate equation of bimolecular charge recombination (CR) reaction can be written as [S2].

$$\frac{\mathrm{d}n(t)}{\mathrm{d}t} = -\gamma_{\mathrm{bi}}(t)\cdot n(t)^2, \qquad \text{(S1)}$$

where γ_{bi} stands for the bimolecular CR rate, and $n(t)$ represents the hole concentration that follows the Beer-Lambert relation

$$n(t) = \Delta\mathrm{OD}(t)\cdot N_A \cdot (1000\cdot\varepsilon_{\mathrm{h}}\cdot d)^{-1}, \qquad \text{(S2)}$$

where N_A is Avogadro's constant, and d and ε_{h} denote the film thickness (190 nm) and the extinction coefficient of hole ($\varepsilon_{\mathrm{h}} = 3 \times 10^4$ $\mathrm{M^{-1}\cdot cm^{-1}}$) [S3], respectively. Combine Equations (1) and (2), we have

$$\gamma_{\mathrm{bi}}(t) = -\frac{\mathrm{d}(\Delta\mathrm{OD}(t))}{\mathrm{d}t}\frac{d\cdot\varepsilon_{\mathrm{P}}}{\Delta\mathrm{OD}(t)^2}\frac{1000}{N_{\mathrm{A}}}. \qquad \text{(S3)}$$

Since the hole dynamics in 70~1500 ps obey the power law, $\Delta\mathrm{OD}(t) = A\cdot t^{-\alpha}$, it follows that

$$\frac{\mathrm{d}(\Delta\mathrm{OD}(t))}{\mathrm{d}t} = -\alpha\cdot t^{-1}\cdot\Delta\mathrm{OD}(t) \qquad \text{(S4)}$$

and

$$\gamma_{\mathrm{bi}}(t) = \frac{1000\cdot\alpha\cdot d\cdot\varepsilon_{\mathrm{h}}}{\Delta\mathrm{OD}(t)\cdot N_{\mathrm{A}}\cdot t}. \qquad \text{(S5)}$$

Following the Langevin hole-limited bimolecular CR model [S4], the hole mobility (μ_{h}) reads

$$\mu_{\mathrm{h}}(t) = \varepsilon_0\cdot\varepsilon\cdot\gamma_{\mathrm{bi}}(t)/e, \qquad \text{(S6)}$$

where ε_0 and ε, respectively, are the vacuum permittivity and the dielectric constant of the P3HT/PC_{61}BM blend, and e is the elementary charge.

S5. Quantitative Analyses of Bimolecular CR Rate and Hole Mobility

Figure S4. (**a**, **b**) Temporal evolution profiles of bimolecular CR rate (γ_{bi}) for the SVA blend P3HT/PC_{61}BM films (1:1, w/w) under photoexcitation at 620 nm or 460 nm. The initial hole concentration at Δt = 70 ps are indicated in each panel. (**c**, **d**) Change of γ_{bi} upon varying the hole concentration at Δt = 70 ps or 1 ns. The excitation wavelengths (λ_{ex}) are indicated in each panel. Solid lines are for guiding the eyes.

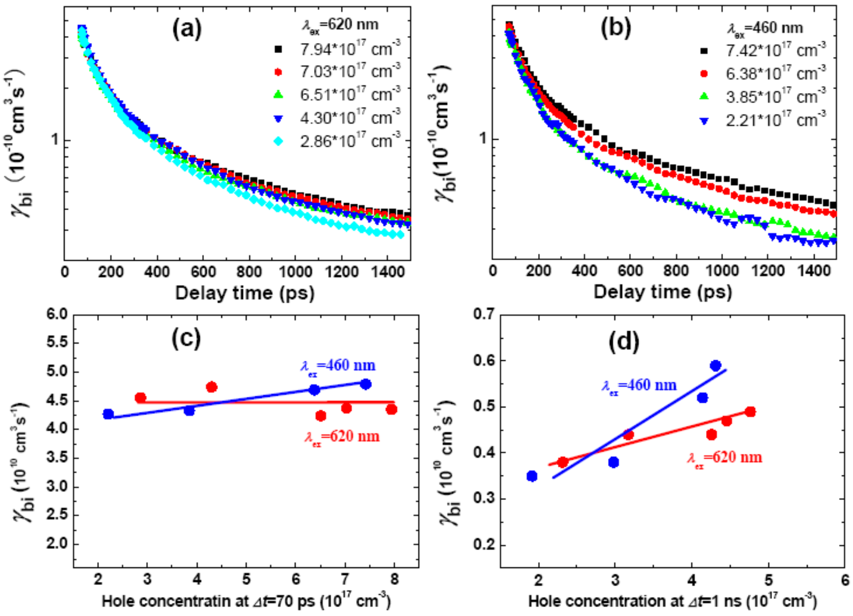

Figure S5. Temporal evolution profiles of bimolecular CR rate (γ_{bi}) and hole mobility (μ_h) for the CS_2-casted blend P3HT/PC_{61}BM films (1:1, w/w) under photoexcitation at 620 nm or 460 nm. The initial hole concentration at Δt = 70 ps are indicated in each panel.

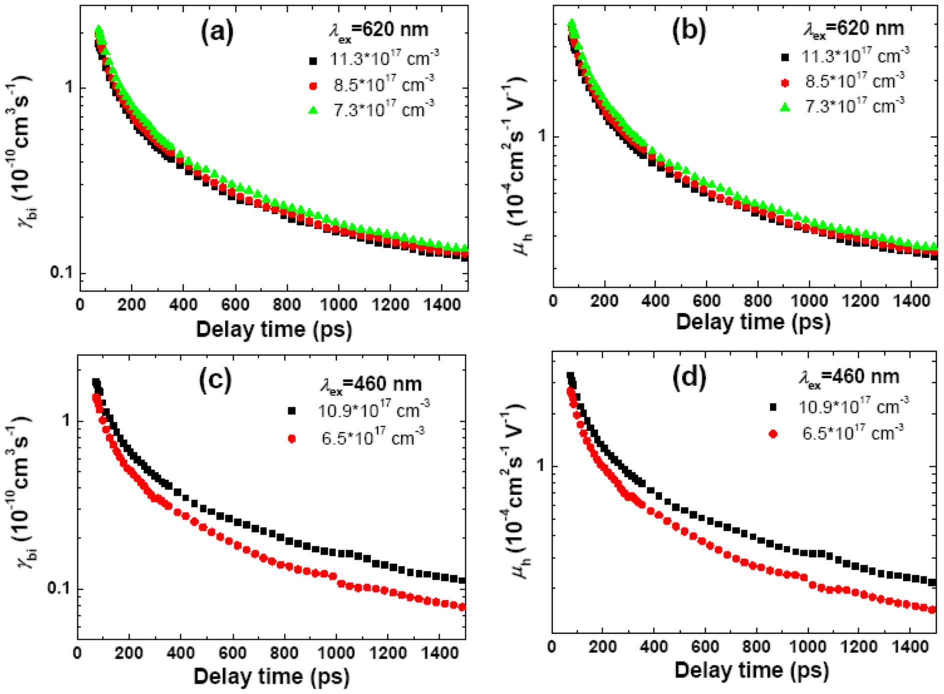

S6. Comparison of Hole mobility to literature values determined with various methods

Table S2 Hole mobility (μ_h) in neat P3HT or blend P3HT/$PC_{61}BM$ films determined by the use of various experimental methods: FET, field effect transistor; TOF, time of flight; CELIV, carrier extraction by linear increase voltage; TRMC, time-resolved microwave conductivity; SCLC, space charge limited current; TA, transient absorption.

Method	Temporal range	μ_h ($cm^2 \cdot V^{-1} \cdot s^{-1}$)	Sample	Reference
FET	steady state	1.7×10^{-6}–9.4×10^{-3} (*a*)	neat p3ht	[S5]
		5×10^{-2} (TiO_2 based)	neat p3ht	[S6]
		1.8×10^{-1} (SiO_2 based)	neat p3ht	[S6]
		1×10^{-2}	neat p3ht	[S7]
TOF	1 μs–1 ms	5.1×10^{-5}	S-p3ht/$PC_{61}BM$ (*b*)	[S8]
	0.1 μs–100 μs	~10^{-4}	T-p3ht/$PC_{61}BM$ (*b*)	[S9]
	0.1 μs–100 μs	1×10^{-4}	neat p3ht	[S10]
CELIV	1 μs–600 μs	10^{-6}–10^{-5} (0.1–10 ms)	RRa-p3ht	[S11]
TRMC	10 ns–450 ns	1.4×10^{-2}	p3ht/$PC_{61}BM$	[S12]
	1 ns–10 μs	5.6×10^{-3} (before)<br>4.4×10^{-2} (after)	p3ht/$PC_{61}BM$<br>T-p3ht/$PC_{61}BM$ (*b*)	[S13]<br>[S13]
SCLC	steady state	2×10^{-8}	T-p3ht/$PC_{61}BM$ (*b*)	[S14]
μs-TA	1 μs–1 ms	10^{-7}–10^{-6}	p3ht/$PC_{61}BM$	[S2]
fs-TA	70 ps–1.5 ns	8.7×10^{-4} ($\Delta t = 70$ ps)	S-p3ht/$PC_{61}BM$ (*b*)	[*this work*]
		8.7×10^{-5} ($\Delta t = 1$ ns)	S-p3ht/$PC_{61}BM$ (*b*)	[*this work*]

[a] Mobility increased from 1.7×10^{-6} to 9.4×10^{-4} $cm^2 \cdot V^{-1} \cdot s^{-1}$ on increasing the molecular weight from 3.2 KD to 36.5 kD; [b] "S" denotes "slow growing" or "solvent annealing" and "T" represents "thermal annealing".

References

S1. Guo, J.; Ohkita, H.; Benten, H.; Ito, S. Near-IR femtosecond transient absorption spectroscopy of ultrafast polaron and triplet exciton formation in polythiophene films with different regioregularities. *J. Am. Chem. Soc.* **2009**, *131*, 16869–16880.

S2. Shuttle, C.G.; O'Regan, B.; Ballantyne, A.M.; Nelson, J.; Bradley, D.D.C.; Durrant, J.R. Bimolecular recombination losses in polythiophene:fullerene solar cells. *Phys. Rev. B* **2008**, *78*, 113201.

S3. Guo, J.; Ohkita, H.; Yokoya, S.; Benten, H.; Ito, S. Bimodal polarons and hole transport in poly(3-hexylthiophene): Fullerene blend films. *J. Am. Chem. Soc.* **2010**, *132*, 9631–9637.

S4. Koster, L.J.A.; Mihailetchi, V.D.; Blom, P.W.M. Bimolecular recombination in polymer/fullerene bulk heterojunction solar cells. *Appl. Phys. Lett.* **2006**, *88*, 052104.

S5. Kline, R.J.; McGehee, M.D. Controlling the field-effect mobility of regioregular polythiophene by changing the molecular weight. *Adv. Mater.* **2003**, *15*, 1519–1522.

S5. Li, Z.Q.; Wang, G.M.; Sai, N.; Moses, D.; Martin, M.C.; Di Ventra, M.; Heeger, A.J.; Basov, D.N. Infrared imaging of the nanometer-thick accumulation layer in organic field-effect transistors. *Nano Lett.* **2006**, *6*, 224–228.

S7. Bürgi, L.; Sirringhaus, H.; Friend, R.H. Noncontact potentiometry of polymer field-effect transistors. *Appl. Phys. Lett.* **2002**, *80*, 2913-2915.

S8. Li, G.; Shrotriya, V.; Huang, J.; Yao, Y.; Moriarty, T.; Emery, K.; Yang, Y. High-efficiency solution processable polymer photovoltaic cells by self-organization of polymer blends. *Nat. Mater.* **2005**, *4*, 864–868.

S9. Kim,Y.; Cook, S.; Tuladhar, S.M.; Choulis, S.A.; Nelson, J.; Durrant, J.R.; Bradley, D.D.C.; Giles, M.; Mcculloch, I.; Ha, C.; *et al.* A strong regioregularity effect in self-organizing conjugated polymer films and high-efficiency polythiophene:fullerene solar cells. *Nat. Mater.* **2006**, *5*, 197–203.

S10. Kim,Y.; Cook, S.; Choulis, S.A.; Nelson, J.; Durrant, J.R.; Bradley, D.D.C. Organic photovoltaic devices based on blends of regioregular poly(3-hexylthiophene) and poly(9,9-dioctylfluorene-*co*-benzothiadiazole). *Chem. Mater.* **2004**, *16*, 4812–4818.

S11. Genevičius, K.; Österbacka, R.; Juška, G.; Arlauskas, K.; Stubb, H. Separation of fast and slow transport in regiorandom poly(3-hexylthiophene). *Synth. Met.* **2003**, *137*, 1407-1408.

S12. Dicker, G.; de Haas, M.P.; Siebbeles, L.D.A.; Warman, J.M. Electrodeless time-resolved microwave conductivity study of charge-carrier photogeneration in regioregular poly(3-hexylthiophene) thin films. *Phys. Rev. B* **2004**, *70*, 045203.

S13 Savenije, T.J.; Kroeze, J.E.; Yang, X.; Loos, J. The formation of crystalline P3HT fibrils upon annealing of a PCBM:P3HT bulk heterojunction. *Thin Solid Films* **2006**, *511*, 2–6.

S14. Mihailetchi, V.D.; Xie, H.; de Boer, B.; Koster, L.J.A.; Blom, P.W.M. Charge transport and photocurrent generation in poly(3-hexylthiophene): Methanofullerene bulk-heterojunction solar cells. *Adv. Funct. Mater.* **2006**, *16*, 699–708.
